# Multiple approaches to reduce reconstitution time of lyophilized drug products with high protein concentration

**DOI:** 10.1093/abt/tbad031

**Published:** 2023-12-29

**Authors:** Xiaozhang Zhang, Ningning Zhou, Chunsheng Yang, Zhaowei Jin, Jeremy Guo

**Affiliations:** Drug Product Development, WuXi Biologics (Shanghai) Co., Ltd, 190 Hedan Road, Waigaoqiao Free Trade Zone, Shanghai, 200131, China; Drug Product Development, WuXi Biologics (Shanghai) Co., Ltd, 190 Hedan Road, Waigaoqiao Free Trade Zone, Shanghai, 200131, China; Drug Product Development, WuXi Biologics (Shanghai) Co., Ltd, 190 Hedan Road, Waigaoqiao Free Trade Zone, Shanghai, 200131, China; Drug Product Development, WuXi Biologics (Shanghai) Co., Ltd, 190 Hedan Road, Waigaoqiao Free Trade Zone, Shanghai, 200131, China; Drug Product Development, WuXi Biologics (Shanghai) Co., Ltd, 190 Hedan Road, Waigaoqiao Free Trade Zone, Shanghai, 200131, China

**Keywords:** lyophilized drug products with high concentration, reconstitution time, multiple approaches, headspace pressure, reducing diluent volume, combined strategy

## Abstract

**Background:**

Lyophilized drug products with high protein concentration often perform long reconstitution time, which is inconvenient for clinical use. The objective of this work is to achieve short reconstitution time with multiple and combined strategies.

**Methods:**

Here, we describe the following approaches that lead to reduction of reconstitution time, including adding annealing step, decreasing headspace pressure, decreasing protein concentration with reducing diluent volume, increasing high surface-area-to-height ratio of the cakes, increasing frequency of swirling and diluent temperature.

**Results:**

Among these strategies, reducing diluent volume to achieve high protein concentration and reducing headspace pressure show markedly reduction of reconstitution time. Moreover, we propose combined strategies to mitigate the reconstitution time, at the same time, to achieve same target dose in clinics.

**Conclusions:**

Therefore, this paper provides insights on the application of multiple strategies to accelerate the reconstitution of lyophilized drug products with high concentration, and facilitates their widespread clinical application.

## Teaser

**Statement of Significance**: Multiple and combined strategies can not only reduce the reconstitution time of lyophilized drug products with high protein concentration, but also will not influence the product quality.

## INTRODUCTION

Drug products with high protein concentration were generally administered by subcutaneous injection (~76% in marketed products), which had the advantages of reducing the treatment time and pain of patients compared with intravenous administration [1]. It has been found that when protein concentration is ˃100 mg/mL, the distance between proteins is reduced to 1–5 nm and hydrophobic interactions become significant, resulting in increased protein aggregation and viscosity, which might bring the challenges for the reconstitution of high-concentration drug products [2]. Drug products with high protein concentration (>50 mg/mL) are often developed into lyophilized form to improve protein stability [3–7]. However, lyophilized drug products with high protein concentration frequently presents the challenge of long reconstitution time [2, 8–11]. The undesirable reconstitution time leads to inconvenience for patient administration, and incomplete protein dissolution prior to use might bring risk of insufficient dosing [12, 13]. Therefore, the long reconstitution time of drug product with high concentration attracts the attention of scientists, and pushes them to overcome this challenge [6, 7, 9, 12].

Scientists made great effort to investigate the root cause of long reconstitution time and to find ways to reduce the time. The factors affecting reconstitution of cake crystallinity and amorphous degree were reported, and Kulkarni *et al.* elucidated that the partial crystallinity in the final cake and larger pore size were identified as desirable characteristics for expediting reconstitution [10, 11]. In addition, scientist added tert-butyl alcohol to the pre-lyophilization formulation and decreased the headspace pressure in the final lyophilized vial in the meantime to reduce reconstitution time [8]. Moreover, reconstitution times of high-concentration lyophilized protein formulations could be improved by adding wetting agents, incorporating annealing steps, reconstituting under vacuum and reduced reconstitution volume [5]. Previous studies had made much contribution to reduce long reconstitution time of freeze-dried drug products with high protein concentration. However, some studies were not well characterized, and combination of multiple methods were not explored. We would like to evaluate the factors in more detailed ways, exploring the methods and conditions that can markedly accelerate reconstitution process, also performing combined strategy, finally giving instruction on the development of lyophilized drug products with high protein concentration.

This paper has evaluated the methods of reducing long reconstitution time from four aspects, including lyophilization process conditions, formulations, vial size and reconstitution methods. Here, high concentration monoclonal antibody (mAb) protein was used in this study. For lyophilization process conditions, incorporating −3°C annealing steps in the lyophilization cycle can reduce by 38% of reconstitution time comparing to nonannealing process. In addition, headspace pressure ˂10 Torr reduced ˃60% of reconstitution time comparing to 250 Torr. For formulations, the reconstitution time was reduced by decrease of protein concentration, and the reduction ratio of reconstitution time was up to 83% with reduced diluent volume to achieve high protein concentration after reconstitution. After evaluating different filling volume in different vial size, it was found that high surface-area-to-height ratio of cake helps to reduce reconstitution time by maximal 46%. In the reconstitution method study, 37°C dilution solution and high-frequency of swirling achieved 56% reduction of reconstitution time. Ultimately, the methods of reducing headspace pressure to ˂10 Torr and reducing diluent volume can significantly reduce the reconstitution time. In addition, combined approaches also provide strategy of mitigating the reconstitution time for drug products. Besides combining other factors, combination group 1 decreased protein concentration to 50 mg/mL, and the overall reduction ratio is 98%. And combination group 2 decreased protein concentration to 75 mg/mL, which can achieve same target formulation after reconstitution and the reduction ratio of 80%. Overall, the above methods provide the multiple strategies to mitigate the reconstitution time and is helpful for clinic use.

## MATERIALS AND METHODS

### Materials

#### Reagents

The mAb (Trastuzumab, Anti HER2 Antibody) was provided by WuXi Biologics. L-histidine monohydrochloride was obtained from Merck. L-histidine and sucrose were gained from Pfanstiehl. Polysorbate 80 (PS80) were purchased from Croda. Type 1 medium borosilicate glass tubing vials (Schott, 2 mL vial, 8 mL vial, 10 mL vial, 20 mL vial, 50 mL vial), stoppers (West, 13 mm, 20 mm) and aluminum covers (West, 13 mm, 20 mm) were used for lyophilization. Syringe of 5 mL and 10 mL were manufactured from Changzhoujinlong Co., Ltd, China.

### Methods

#### Information of formulations

The protein formulations contained histidine buffer at pH 6.0. All the formulations contained 20 mM histidine, 8% (w/v) sucrose and 0.02% (w/v) polysorbate 80 surfactant, except F3 contained 10 mM histidine, 4% (w/v) sucrose and 0.01% (w/v) PS80. For protein concentration, F1, F2, F3, F4 and F5 were formulated with 150, 75, 75, 100 and 50 mg/mL protein, respectively. Table 1 summarizes the information of formulations.

**Table 1:** Information of formulations

No.	Protein con. (mg/mL)	Protein amount/mg	Formulation	Filling volume/mL	Reconstitution volume/mL
F1	150	225	20 mM Histidine, 8%(w/v) sucrose, 0.02%(w/v) PS80	1.5	1.5
F2	75	225	20 mM Histidine, 8%(w/v) sucrose, 0.02%(w/v) PS80	3	3
F3	75	225	10 mM Histidine, 4%(w/v) sucrose, 0.01%(w/v) PS80	3	1.5
F4	100	150	20 mM Histidine, 8%(w/v) sucrose, 0.02%(w/v) PS80	1.5	1.5
F5	50	75	20 mM Histidine, 8%(w/v) sucrose, 0.02%(w/v) PS80	1.5	1.5

#### Lyophilization process

##### Cycle 1: Nonannealing process

The vials were lyophilized in a Tofflon LYO-0.5 freeze-dryer. Vials were loaded onto the shelf at temperature (Ts) of 5°C, and held for 30 min before cooling with 1°C/min to −5°C. After holding for 60 min to provide partial thermal equilibration in the solutions, Ts was then cooled to −45°C at 1°C/min and held for 3 h. For primary drying, the chamber vacuum was lowered to 100 mTorr and the Ts was raised to the shelf set point of −10°C at 1°C/min and held for 40 h. Then the Ts was ramped to 35°C for secondary drying at the rate of 0.5°C/min and held for 10 h.

##### Cycle 2: Annealing process

During freezing steps, samples were cooled to 5°C and held for 30 min and then cooled with 1°C/min to −5°C. After holding for 60 min, the shelf was then cooled to −45°C at 1°C/min and held for 3 h. Annealing step was performed with Ts raising to −3°C, −10°C or − 15°C using a ramp rate of 1°C/min and holding for 3 h. After that, Ts was cooled back to −45°C at a ramp rate of 1°C/min and held for 3 h. The subsequent drying steps were same with nonannealing process.

##### Backfill and stoppering

After lyophilization, vials were backfilled with different vacuum (0.1 Torr, 10 Torr, 50 Torr, 100 Torr and 250 Torr) by nitrogen gas to evaluate the effect of headspace vacuum on reconstitution time. Then, the vials were fully stoppered.

#### Scanning electron microscopy

Scanning electron microscopy (SEM) images of the lyophilized cakes have been acquired on a Phenom XL from Phenom Scientific (Netherlands). The backscattered electron detector with acceleration voltage of 5 kV was used under low vacuum of 60 Pa to reduce surface charge on the sample during image acquisition. Line averaging of 20 scans per frame was applied to reduce noise, resulting in a full acquisition time of 2 s per image. In order to allow good conductivity, the samples were gold sputtered using the ISC150 from SuPro (China). The parameters for sputtering were set to 120 s with a power of 10 W under a flow of argon at 6 bar.

#### Specific surface area—Brunauer–Emmett–Teller

The lyophilized cake surface area was determined using nitrogen gas adsorption on an Autosorb iQ Station 1- (Quantachrome Instruments, Boynton Beach, FL, USA). Samples were degassed for at least 3 h. The instrument was calibrated using 100% nitrogen gas at room temperature and at the pressure recorded during the time of measurement. Adsorption volumes were determined at multiple points from a 0.05 to 0.2 p/p0 pressure range at 77.4 K.

#### Cake resistance to crushing

The material mechanics tester (Model 2519-203, Instron Corporation, Norwood, MA, USA) can quantitatively record the resistance of the cake to crushing in the dry state. The force used when the cylindrical probe was inserted vertically into different depths of cake. In this experiment, diameter of stainless-steel cylindrical probe is 2.9 mm and constant rate is 0.5 mm/min. The cylindrical probe was attached to a calibrated 100 N load cell. The vials were decapped and placed on the sample stage. The freeze-dried cake was equilibrated at room temperature before being tested. The test was terminated when the probe displacement was 1.5 mm into the cake. The force versus displacement of four replicate samples was recorded.

#### HIAC

A liquid particle counting system (Model HIAC 9703+, HACH, USA) was utilized to measure the size and number of sub-visible particles in a bio-safety hood. To avoid introducing air bubbles and interference during examination, all samples were held in the bio-safety hood for at least 0.5 h before testing. Each sample was tested four consecutive runs, 0.45 mL for each run. First run was discarded and results were presented as average number of particles ≥10 μm and ≥25 μm per mL from last three runs (method conforms to United States Pharmacopoeia (USP) <788> Particulate matter in injections).

#### Size-exclusion chromatography

Size-Exclusion Ultra-Performance-Liquid-Chromatography (SE_UPLC)(Model 1290, Agilent Technologies, Inc., Santa Clara, CA, USA) is a purity analysis method that separates proteins based on their size. The procedure of SE-UPLC analysis is as follows: the sample was diluted to 10 mg/mL with the mobile phase (50 mM phosphate buffer, 300 mM sodium chloride, 100 mM L-arginine and 10% isopropanol (IPA), pH 6.8 ± 0.1) before SE-UPLC analysis if the sample was above 10 mg/mL. For each sample, 100 μg of protein was injected into the Agilent 1260 UPLC system with a TSKgel G3000SWXL column (7.8 × 300 mm, 5 μm particle size) and a UV detector (detection wavelength: 280 nm). An isocratic gradient was applied for 20 min at a flow rate of 0.8 mL/min with next injection delay of 10 min. All the raw data were processed with Empower 3.

#### Caliper-Sodium Dodecyl Sulfate (SDS)-NR & R

Caliper-SDS (Model Maurice C, ProteinSimple Instruments, CA, USA) was performed on a PerkinElmer Caliper automated electrophoresis system. Firstly, the sample denaturing solution was prepared by mixing sample buffer with 10% SDS and 100 mM N-ethylmaleimide (NEM) (for reduced, 1 M dithiothreitol) at the volume ratio of 20:1:0.735. Before running, diluted samples and diluted reference standard was mixed with the sample denaturing solution. In addition, protein ladder (supplied with the Caliper Reagent Kit) was prepared, which were incubated at 70°C for 10 min and then mixed. Then, water was added to each sample and blank control vial, and all the vials were vortexed, centrifuged and transferred individually to a 96-well plate. After that, the sample plate was placed onto a Labchip GXII’s plate holder, prepared samples and control solutions were loaded, stained, separated and detected in the HT Protein Express LabChip filled with destain-gel, gel-dye and marker. At last, the raw data were analyzed with LabChip GX Reviewer software.

#### Reconstitution procedure

Reconstitution behaviors of 10 vials of each formulation were characterized. At the end of the lyophilization, the samples were rewarmed to room temperature and reconstitution immediately using Milli-Q Water (at room temperature). Reconstitution solvent was injected into the vial in ˂10 s. Swirling of the vial around a circle with diameter of 7.5 cm. Two different swirling frequencies were designed in the experiment: high-frequency swirling and low-frequency swirling. The high-frequency swirling procedure: after injection of the Milli-Q water, the vial was swirled with 75 revolution per minute (rpm) for 3 min and kept upright for 1 min for observation, then it was swirled with 75 rpm again. The procedure was repeated until the solid is totally dissolved. The low-frequency swirling procedure: similarly, after injection of the Milli-Q water, the vial was swirled with 48 rpm for 3 min and stand for 1 min for observation. The procedure was repeated until the solid is totally dissolved, which absence of visible cake or powder indicated the end point of reconstitution. This procedure was used to ensure consistent operation between analysts. Reconstitution time was recorded once the needle was inserted into the vial, including swirling time and stop observation time. In addition, reduction ratio is defined as follow:


$${{\begin{align*} &\mathrm{Reduction}\ \mathrm{ratio}=\\&\frac{\mathrm{Reconstitution}\ \mathrm{time}\ \mathrm{of}\ \mathrm{control}\ \mathrm{group}-\mathrm{Reconstitution}\ \mathrm{time}\ \mathrm{of}\ \mathrm{experiment}\ \mathrm{group}}{\mathrm{Reconstitution}\ \mathrm{time}\ \mathrm{of}\ \mathrm{control}\ \mathrm{group}\mathrm{s}}\% \end{align*}}}$$

## RESULTS AND DISCUSSION

### Lyophilization process conditions

#### Annealing process

**Figure 1:**
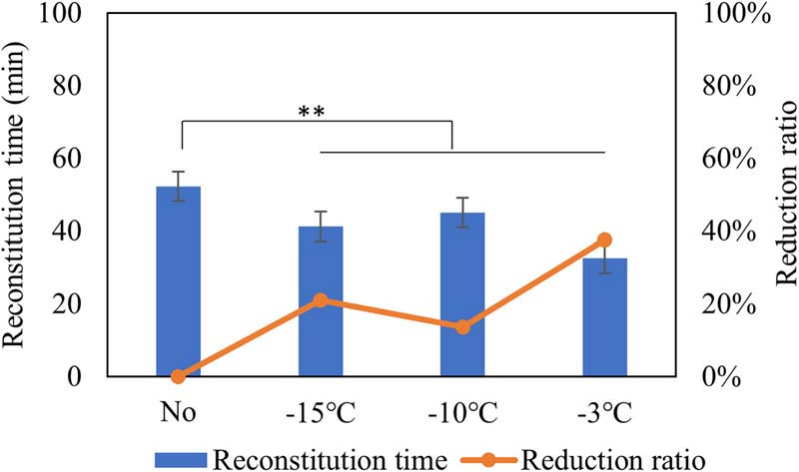
Reconstitution time and reduction ratio of lyophilized formulations (F1, 150 mg/mL protein concentration) without or with annealing process. Formulations in this study were reconstituted following the low-frequency swirling procedure. *n* = 10 for each analysis. Statistically significant differences between the non-annealing and annealing cakes are denoted by ^*^^*^(*P* < 0.01).

In general, larger pores aid penetration of the reconstitution fluid and reduce reconstitution time even in the presence of foam [10, 11, 14–16]. This can be achieved by controlling ice nucleation at higher temperatures [13, 17]. Both time and temperature of annealing are significant in forming the porous structure of cakes containing highly concentrated proteins [18]. Figure 1 shows the comparison of reconstitution time of cakes with or without annealing step in the freezing stage. Annealing temperature was set as −3°C, −10°C and − 15°C in the experiment and the process without annealing process was used as control. Filling volume of 3 mL in 8 mL vial was performed in this study. Results show that annealing steps had apparently positive effect on the reconstitution time reduction of high protein concentration formulation. 21% and 14% reduction in reconstitution time for samples annealed at −15°C and −10°C were observed, respectively. And 38% reduction for samples annealed at −3°C were observed compared to nonannealing process.

**Figure 2:**
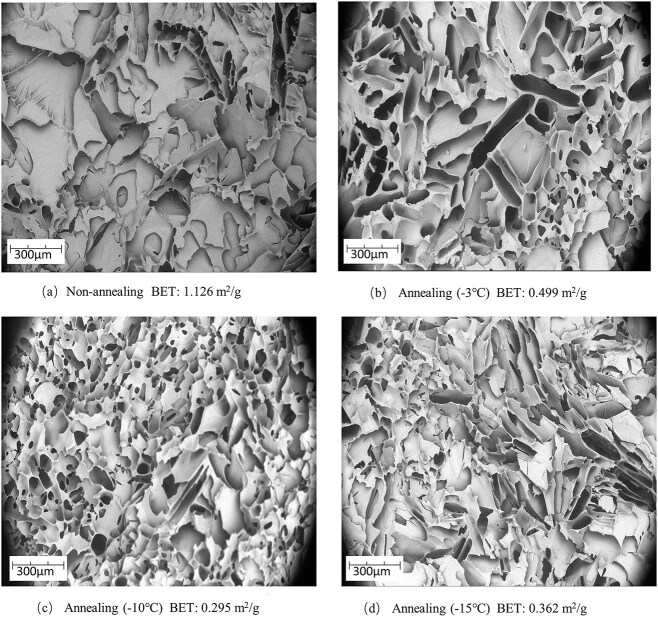
SEM images of freeze-dried cakes obtained from (a) non-annealing, (b) annealing (−3°C), (c) annealing (−10°C) and (d) annealing (−15°C) process. Numbers below images represent specific surface area obtained by Brunauer–Emmett–Teller.

**Figure 3:**
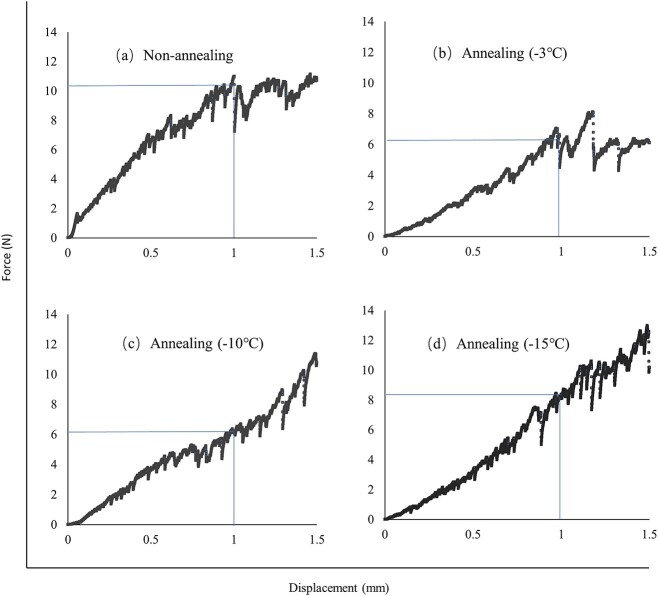
Representative force vs. displacement curves for freeze-dried cakes obtained from (a) non-annealing, (b) annealing (−3°C), (c) annealing (−10°C) and (d) annealing (−15°C).

In addition, SEM images, specific surface area and crushing resistance was further performed to indicate that adding the annealing process can facilitated reconstitution [19–22]. From Figure 2, firstly, the cake having undergone non-annealing process shows smaller pore size than annealing process. However, no significant difference was observed when annealing temperature was set as −3°C, −10°C and − 15°C by SEM images. Secondly, non-annealing process shows cake with lager specific surface area is obtained compared with annealing process. From Figure 3, annealing process in the cake crushed more easily than non-annealing process, which indicates larger pore size and loose structure during annealing process. Overall, it is speculated that all aspects of larger pore size, smaller specific surface area and loose structure of cake facilitate penetration of reconstituted fluid, and then help reconstitution.

#### Headspace pressure

**Figure 4:**
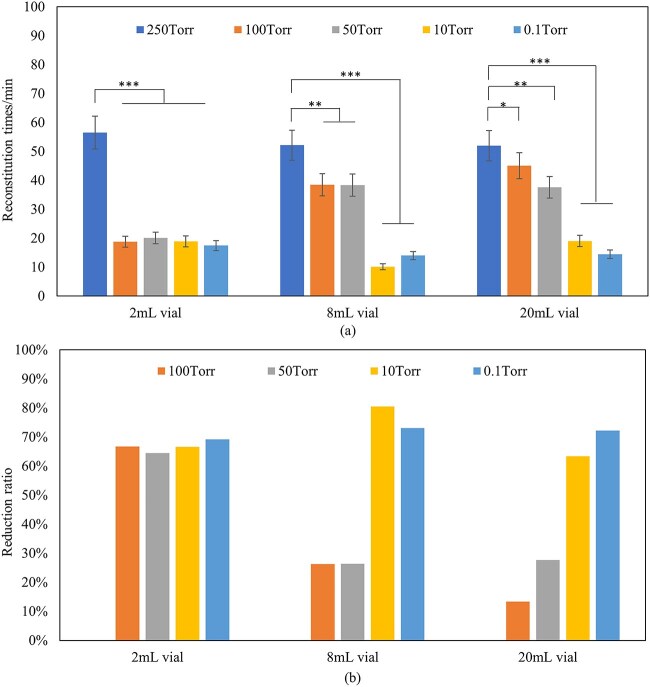
Reconstitution time (a) and reduction ratio (b) of lyophilized formulations (F1,150 mg/mL protein concentration) with 225 mg protein in 2 mL vial, 450 mg in 8 mL vial and 900 mg in 20 mL vial at headspace vacuum range from 250 Torr to 0.1 Torr. Formulations in this study were reconstituted following the low-frequency swirling procedure. *n* = 10 for each analysis. Error bars represent one standard deviation. Statistically significant differences between the 250 Torr, 100 Torr, 50 Torr, 10 Torr and 0.1 Torr cakes are denoted by ^*^(*P* < 0.05), ^*^^*^(*P* < 0.01) and ^*^^*^^*^(*P* < 0.001).

The relationship between reconstitution time and headspace pressure were evaluated in different vial sizes. Figure 4a shows the reconstitution time with filling volume of 1.5 mL in 2 mL vial, 3 mL in 8 mL vial and 6 mL in 20 mL vial under 250 Torr, 100 Torr, 50 Torr, 10 Torr, 0.1 Torr headspace vacuum, and Figure 4b shows the time reduction ratio under 100 Torr, 50 Torr, 10 Torr, 0.1 Torr compared with 250 Torr. Products with lower headspace pressure in the vial shows short reconstitution time compared to higher headspace pressure. In 2 mL vials, samples with headspace pressure ˂100 Torr shows great decrease of reconstitution time, and 69% reduction in reconstitution time was observed with reconstitution time of 17 min at 0.1 Torr compared with 56 min at 250 Torr. In 8 mL vials, samples with headspace pressure ˂10 Torr shows great decrease of reconstitution time, and 81% reduction in reconstitution time was observed with reconstitution time of 10 min at 10 Torr compared with 52 min at the 250 Torr. In 20 mL vials, samples with headspace pressure ˂0.1 Torr shows great decrease of reconstitution time, and 72% reduction in reconstitution time was observed with reconstitution time of 14 min at the 0.1 Torr compared with 52 min at the 250 Torr. Therefore, reconstitution time depends on the headspace pressure and the vial size. It is speculated that sample in larger vial size may require lower headspace pressure to achieve similar reconstitution time reduction as that in smaller vials. The increased permeability of the diluent into the cake may be responsible for the observed phenomenon, as the lower pressure means that there is less gas in the pores blocking the flow of the diluent.

### Formulations

**Figure 5:**
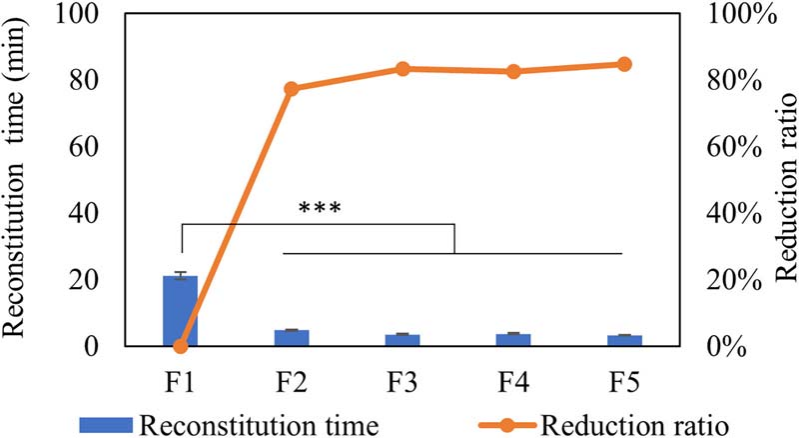
Reconstitution time and reduction ratio of lyophilized formulations with protein concentrations range from 150 to 50 mg/mL (protein amount range from 225 to 75 mg). Formulations in this study were reconstituted following the low-frequency swirling procedure. *n* = 10 for each analysis. Error bars represent one standard deviation. Statistically significant differences between the F1, F2, F3, F4 and F5 cakes are denoted by ^*^^*^^*^(*P* < 0.001).

Effect of protein concentration was studied with filling volume of 1.5 mL and 3 mL in 8 mL vials under nonannealing lyophilization process. Five formulations ranged from 50 mg/mL to 150 mg/mL are listed in Table 1, and F1 is regarded as control. Figure 5 shows the reconstitution time of lyophilized formulations with different protein concentrations. With the protein concentration decreased from 150 to 50 mg/mL (F1, F4 and F5), there was significant decrease in reconstitution time from 21 to 3 min, which indicating reconstitution time was greatly influenced by protein concentration. In addition, by comparing F1 and F3 with same protein amount, it was found that reconstitution time of F1 can be greatly reduced by freeze-drying formulation with half concentration and 2-fold filling volume, then reconstituting with half of filling volume (F3) to achieve same target protein concentration and formulations. Another method to reduce reconstitution time with same protein amount is also to freeze-dry formulation with half concentration and two-fold filling volume while reconstituting with same filling volume (F2). Therefore, F2 and F3 can be excellent choices for formulation development of lyophilized drug products with high protein concentration with purpose of reducing reconstitution time.

### Vial size—Surface area to height ratio of the lyophilized cake

**Figure 6:**
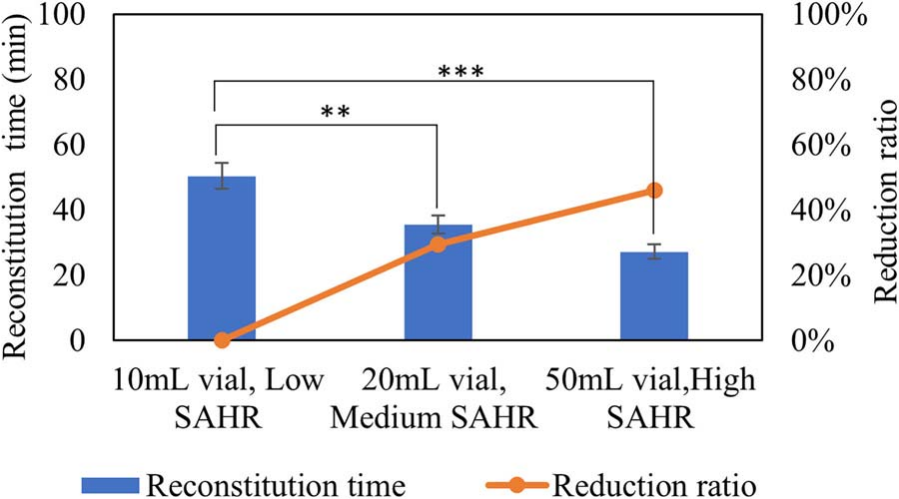
Reconstitution time and reduction ratio of lyophilized formulations (F1, 150 mg/mL protein concentration) with 600 mg protein in 10 mL vial, 20 mL vial and 50 mL vial. Formulations in this study were reconstituted following the low-frequency swirling procedure. *n* = 10 for each analysis. Error bars represent one standard deviation. Statistically significant differences between the low SAHR, medium SAHR and high SAHR cakes are denoted by ^*^^*^(*P* < 0.01) and ^*^^*^^*^(*P* < 0.001).

As we known, the surface area and height of cake in the vials are different if same volume of solution are filled into different vials. Table 2 shows the reconstitution time and reduction ratio with different surface area to height ratio (SAHR) of the lyophilized cakes in different vials (filling volume of 4 mL in 10 mL, 20 mL, 50 mL vial). Figure 6 shows that high SAHR of lyophilized cake has more significant reduction ratio compared with control group, which also provides useful strategy for vial size selection in development of high concentrated lyophilized drug products with long reconstitution time.

**Table 2:** Reconstitution time and reduction ratio with different surface area to height ratio of the lyophilized cake in different vials

Vial	Filling volume (mL)	Filling surface area (cm^2^)	Filling height (cm)	SAHR	Reconstitution time (min)	Reduction ratio
10 mL vial, Low SAHR	4.0	3.8	1.05	3.6	50.4	Control
20 mL vial, Medium SAHR	4.0	6.0	0.67	8.9	35.5	30%
50 mL vial, High SAHR	4.0	10.8	0.37	29.1	27.2	46%

### Reconstitution methods

#### Frequency of swirling and diluent temperature

**Figure 7:**
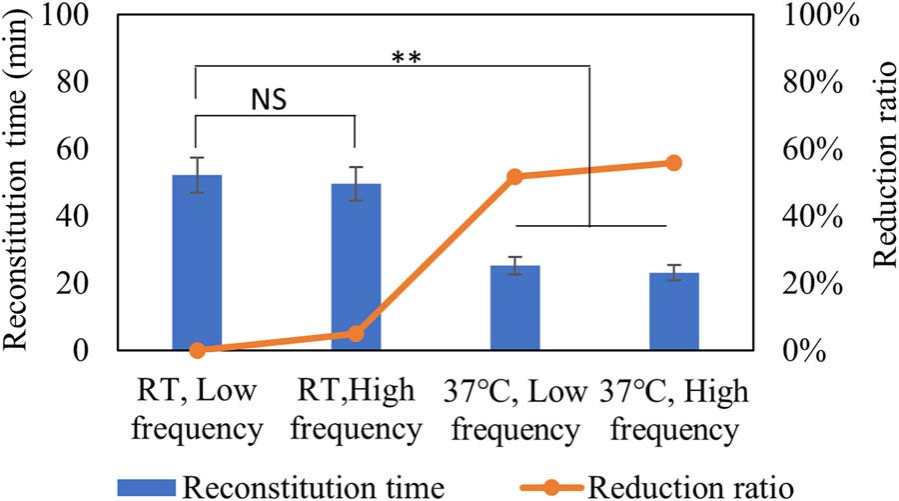
Reconstitution time and reduction ratio of lyophilized formulations (F1, 150 mg/mL protein concentration) with 450 mg protein in 8 mL vial. Reconstitution for frequency of swirling from low to high and diluent temperature from room temperature to 37°C. *n* = 10 for each analysis. Error bars represent one standard deviation. Statistically significant differences between the RT-low frequency, RT-high frequency, 37°C-low frequency and 37°C-high frequency cakes are denoted by NS (*P* ˃ 0.05) and ^*^^*^(*P* < 0.01).

This part shows that the diluent temperature and frequency of swirling are significant and direct factors to affect reconstitution time. In this study, 3 mL formulation liquid with protein concentration of 150 mg/mL was filled in 8 mL vial, then lyophilized without annealing step. Figure 7 shows the reduction of reconstitution time under different diluent temperature (room temperature and 37°C) and frequency of swirling (low and high frequency). 37°C with high frequency of swirling had a significant effect on reconstitution time. This result indicates that palm-warming of diluent before reconstitution and higher swirling frequency will be benefit for reconstitution.

### Combined method

**Figure 8:**
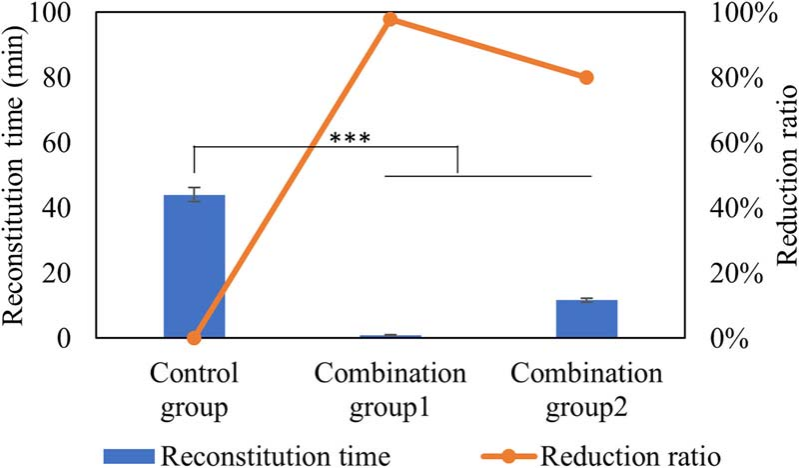
Reconstitution time and reduction ratio of lyophilized formulations for combination group. *n* = 10 for each analysis. Error bars represent one standard deviation. Statistically significant differences between the Control group, combination group1 and combination group2 cakes are denoted by ^*^^*^^*^ (*P* < 0.001).

According to the above results, we found that annealing process, headspace pressure, formulations, container size and reconstitution methods show effect on the reconstitution time. Here, we propose combined approaches to provide strategy for mitigating the reconstitution time for lyophilized drug products with high protein concentration. Table 3 shows the experimental condition of combination groups, and Figure 8 shows the reconstitution time and reduction ratio. Compared with control group, both of combination group 1 and group 2 decreased the protein concentration, increased the surface-area-to-height ratio, adopted annealing process with high temperature and low headspace pressure, and reconstituted with high-swirling frequency and temperature. The difference of group 1 and group 2 was the formulation and surface-area-to-height ratio (4.2 and 5.4). Group 1 decreased protein concentration to 50 mg/mL, and shows significant reduction of reconstitution time overall (98%). This method required three vials (150 mg/vial) to achieve same target dose as control group (450 mg). In addition, group 2 freeze-dried formulation with half concentration and 2-fold fill volume than control group, then reconstituted with half of fill volume. The overall reduction ratio was 80%. This method can achieve same target formulation and target dose (450 mg/vial) compared with control group after reconstitution. Therefore, both of these two combination groups can significantly reduce the reconstitution time. Moreover, comprehensive consideration is required in different reconstitution strategies, such as users’ convenience, manufacturing process time and lyophilization cycle time to balance the benefit and the cost.

**Table 3:** The experimental condition of combination groups

Factors	Control	Combination Group 1	Combination Group 2
Formulation	F1	F5	F3
Surface-area-to-height ratio	8 mL vial (Filling volume: 3 mL), SAHR: 2.9	10 mL vial (Filling volume: 3 mL), SAHR: 4.2	20 mL vial (Filling volume: 6 mL), SAHR: 5.4
Annealing process	Nonannealing process	Annealing (−3°C)	Annealing (−3°C)
Headspace vacuum	250 Torr	10 Torr	10 Torr
Frequency of swirling and diluent temperature	Gentle swirling for 1 rps, room temperature	Gentle swirling for 2 rps, 37°C	Gentle swirling for 2 rps, 37°C

The stability of control group and combination groups are shown in Table 4. No substantial changes of Size Exclusion Chromatography (SEC) monomer% and purity% of SDS caliper were observed for combination groups compared with control group. In addition, no substantial changes of sub-visible particles were found in combination group compared with control group, and the sub-visible particles of all the groups were within the acceptable range in USP <788>. The results indicate that the combined method will not influence the protein stability.

**Table 4:** SE-UPLC and SDS-Caliper-NR&R results for control group and combination group after reconstitution

Formulation	SE-UPLC			SDS-Caliper-NR	SDS-Caliper-R	Sub-visible particle Counts (#/mL)	
	Monomer%	HMW%	LMW%	Purity%	Purity%	≥10 μm	≥25 μm
Control group	94.6	5.3	0.2	96.8	99.3	169	2
Combination group1	94.7	5.1	0.2	96.8	99.3	194	0
Combination group2	94.5	5.3	0.2	96.8	99.3	754	19

## CONCLUSIONS

Reconstitution time is an important product quality attribute for lyophilized drug products, especially with high protein concentration. However, long reconstitution time is a challenging issue, which brings about inconvenience of drug products for clinical use. This study highlighted evaluation of variable factors influencing the reconstitution time, such as lyophilization process, formulations, container size and reconstitution methods. Methods that can significantly speed up reconstitution process were also pointed out, and the combined methods were proposed. In terms of protein concentration, 83% reduction of reconstitution time was achieved by lyophilization of lower concentration formulation with higher filling volume and reconstituting with reduced diluent volume. In the aspect of lyophilization process, ˃60% reduction in reconstitution time was observed at ˂10 Torr headspace pressure compared with 250 Torr. In addition, 38% reduction in reconstitution time was observed when annealing at −3°C compared with nonannealing process. Moreover, in consideration of container size, higher SAHR of lyophilized cake had more significant reduction ratio. Besides, reconstitution methods such as high diluent temperature and high frequency of swirling were helpful for reconstitution. Particularly, combined approaches with all factors above can also be selected as strategy of reducing the reconstitution time for drug products, which achieved reduction ratio of 98% as the best with little potential impact on protein quality. Overall, multiple and combined strategies to solve the reconstitution problem are proposed in this study, and this may facilitate the wide application of lyophilized drug products with high protein concentration.

## Supplementary Material

Supplementary_Data_tbad031

## Data Availability

Data are available in the Supplementary Material and upon reasonable request to the corresponding author.
