# Supplementary material for: Multiple approaches to reduce reconstitution time of lyophilized drug products with high protein concentration
Source: Antib Ther. 2023 Dec 29;7(1):67–76. doi: 10.1093/abt/tbad031 (PMC10873283; doi:10.1093/abt/tbad031)
Supplement: Supplementary_Data_tbad031 [file supplementary_data_tbad031.docx]

**Multiple Approaches to Reduce Reconstitution Time of Lyophilized Drug Products with High Protein Concentration**

*Xiaozhang Zhang, Ningning Zhou, Chunsheng Yang, Zhaowei Jin, Jeremy Guo**

Drug Product Development, WuXi Biologics (Shanghai) Co., Ltd., Shanghai, China

**Supplementary data**

**Table S1** Reconstitution time and reduction ratio of formulations at different conditions

| Annealing process | | | | | |
| --- | --- | --- | --- | --- | --- |
| Annealing temperature (℃) | Formulation | Vial (mL) | Filling volume (mL) | Reconstitution time (min) | Reduction ratio |
| No | F1 | 8 | 3 | 52 | 0 |
| -15 | F1 | 8 | 3 | 41 | 21% |
| -10 | F1 | 8 | 3 | 45 | 14% |
| -3 | F1 | 8 | 3 | 33 | 38% |
| Headspace pressure | | | | | |
| Headspace pressure (Torr) | Formulation | Vial (mL) | Filling volume (mL) | Reconstitution time (min) | Reduction ratio |
| 250 | F1 | 2 | 1.5 | 57 | 0 |
|  | F1 | 8 | 3 | 52 | 0 |
|  | F1 | 20 | 6 | 52 | 0 |
| 100 | F1 | 2 | 1.5 | 19 | 67% |
|  | F1 | 8 | 3 | 38 | 26% |
|  | F1 | 20 | 6 | 45 | 13% |
| 50 | F1 | 2 | 1.5 | 20 | 64% |
|  | F1 | 8 | 3 | 38 | 26% |
|  | F1 | 20 | 6 | 38 | 28% |
| 10 | F1 | 2 | 1.5 | 19 | 67% |
|  | F1 | 8 | 3 | 10 | 81% |
|  | F1 | 20 | 6 | 19 | 63% |
| 0.1 | F1 | 2 | 1.5 | 17 | 69% |
|  | F1 | 8 | 3 | 14 | 73% |
|  | F1 | 20 | 6 | 14 | 72% |
| Formulations | | | | | |
| Protein Con.(mg/mL) | Formulation | Vial (mL) | Filling volume (mL) | Reconstitution time (min) | Reduction ratio |
| 150 | F1 | 8 | 1.5 | 21 | 0 |
| 75 | F2 | 8 | 3 | 5 | 77% |
| 75 | F3 | 8 | 3 | 4 | 83% |
| 100 | F4 | 8 | 1.5 | 4 | 82% |
| 50 | F5 | 8 | 1.5 | 3 | 85% |
| Vial Size | | | | | |
| SAHR | Formulation | Vial (mL) | Filling volume (mL) | Reconstitution time (min) | Reduction ratio |
| Low | F1 | 10 | 4 | 50 | 0 |
| Medium | F1 | 20 | 4 | 36 | 30% |
| High | F1 | 50 | 4 | 27 | 46% |
| Reconstitution Methods | | | | | |
| Frequency of swirling and diluent temperature | Formulation | Vial (mL) | Filling volume (mL) | Reconstitution time (min) | Reduction ratio |
| Room temperature, Low frequency | F1 | 8 | 3 | 52 | 0 |
| Room temperature, High frequency | F1 | 8 | 3 | 50 | 5% |
| 37℃, Low frequency | F1 | 8 | 3 | 25 | 52% |
| 37℃, High frequency | F1 | 8 | 3 | 23 | 56% |
| Combined Methods | | | | | |
| Group | Formulation | Vial (mL) | Filling volume (mL) | Reconstitution time (min) | Reduction ratio |
| Control | F1 | 8 | 3 | 52 | 0 |
| Combination Group 1 | F1 | 10 | 3 | 1 | 98% |
| Combination Group 2 | F1 | 20 | 6 | 10 | 80% |
